# Impact of Recycler Information Sharing on Supply Chain Performance of Construction and Demolition Waste Resource Utilization

**DOI:** 10.3390/ijerph19073878

**Published:** 2022-03-24

**Authors:** Haoxuan Zheng, Xingwei Li, Xiaowen Zhu, Yicheng Huang, Zhili Liu, Yuxin Liu, Jiaxin Liu, Xiangye Li, Yuejia Li, Chunhui Li

**Affiliations:** 1 College of Architecture and Urban-Rural Planning, Sichuan Agricultural University, Chengdu 611830, China; 201908323@stu.sicau.edu.cn (H.Z.); huangyicheng@stu.sicau.edu.cn (Y.H.); 201908355@stu.sicau.edu.cn (Z.L.); 202008217@stu.sicau.edu.cn (Y.L.); 202008216@stu.sicau.edu.cn (J.L.); 202008214@stu.sicau.edu.cn (X.L.); 202008215@stu.sicau.edu.cn (Y.L.); 202008213@stu.sicau.edu.cn (C.L.); 2 School of Management, Jiangsu University, Zhenjiang 212013, China; xiaowen.zhu@cranfield.ac.uk; 3 Centre for Design, Cranfield University, Cranfield, Bedfordshire MK43 0AL, UK

**Keywords:** supply chain performance, dual-channel reverse supply chain, construction and demolition waste (CDW), Stackelberg game, information sharing

## Abstract

In recent years, the generation of a large amount of construction and demolition waste (CDW) has threatened the public environment and human health. The inefficient supply chain of CDW resource utilization hinders the green development of countries around the world, including China. This study aims to reveal the impact of information sharing regarding recyclers’ market demand forecast on the performance of CDW resource utilization supply chains. Therefore, this paper uses the incomplete information dynamic game method to establish and solve the decision-making model of the construction and demolition waste resource utilization supply chain under the conditions of recyclers sharing and not sharing their information. The paper then obtains the Bayesian equilibrium solution and the optimal expected profit for each party. Finally, a numerical simulation was used in order to verify the validity of the model and conclusions. The main conclusions are as follows. In the CDW resource utilization supply chain, if the recycler is more pessimistic about the market’s demand forecast, their information sharing makes the remanufacturer more motivated to improve their level of environmental responsibility. In addition, information sharing by recyclers is always beneficial in increasing the profit of the remanufacturer, but it also may make the recycler lose profit. When the efficiency of the environmental responsibility investment of remanufacturers is in a high range, information sharing increases the profits of recyclers. Conversely, information sharing has no significant effect on the profits of recyclers. The impact on the profits of the entire CDW resource utilization supply chain depends on the intensity of competition among channels, the market share of offline recycling channels and the efficiency of environmental responsibility investments.

## 1. Introduction

Over the past few decades, the massive production of construction and demolition waste (CDW) is increasingly threatening the public environment and human health globally [1]. Although people have gradually realized the urgency of promoting the recycling of construction and demolition waste, the development of CDW treatment is not well developed in China [2]. Existing research indicates that the relatively low resource utilization of CDW in China is due to the lack of a developed CDW recycling market [3]. Additionally, the fairly low level of development of the CDW recycling market suggests that considerable time and money must be invested in building relationships and tracking pricing changes [4]. However, the difficulty of building collaborative relationships has been a problem for enterprises, hindering their efforts to improve their overall performance [5]. The resulting impact has made it difficult to achieve the high-quality development of CDW resource utilization supply chains. In China, CDW accounts for about 30–40% of the total amount of urban waste, while its resource utilization rate is less than 5%; but in developed countries such as Japan and the European Union, its utilization has exceeded 90% [6,7].

In recent years, various issues that are related to CDW management have attracted extensive academic attention. Many scholars have studied the properties of CDW itself and those of the remanufactured products that are produced from construction and demolition waste [8,9,10,11,12]. Some scholars have also studied the application of green development concepts such as circular economy theory, 3R principles and whole life cycle strategies in CDW management [2,13,14]. While these academics have made significant contributions to the development of CDW management, there are still some problems that are yet to be resolved. In the construction field, remanufacturers recycle CDW both directly from building material consumers and indirectly from CDW recyclers. As a result, there is both a cooperative relationship and a conflicting relationship between the remanufacturer and the CDW recycler, between channels.

The management of this channel conflict affects the success of dual-channel supply chains [15]. Channel conflict among channel members tends to be a very negative force and will result in the less efficient recycling of construction and demolition waste. If we cannot eliminate the problem of channel conflict, it will be difficult to improve the efficiency of CDW recycling. Furthermore, the dual-channel reverse supply chain focuses on the integration of logistics, capital flow and information flow on the reverse chain, which is conducive to helping enterprises to overcome the conflicts between channels. Therefore, the concept of dual-channel reverse supply chain management is very suitable for CDW management. However, few scholars have optimized the CDW management model from the perspective of a dual-channel reverse supply chain operation.

Information sharing is widely recognized as an important tool for coordinating supply chain activities, a key factor in reducing supply chain costs and a major means of improving supply chain performance [16,17,18]. In the construction field, the uncertainty of market demand significantly increases the decision risk for supply chain stakeholders. Therefore, it is particularly important to forecast market demand information. As the CDW recycler is closer to the consumer market, their market information can be obtained in order to more effectively predict the market demand. Effective information sharing will significantly enhance the effectiveness of supply chain practices and further contribute to good supply chain performance [19,20,21]. Therefore, information sharing by the recycler should be considered in the decision-making process of the CDW resource utilization supply chain.

Therefore, studying the impact of recyclers’ information sharing on the performance of CDW resource utilization supply chain is of great significance for the development of a circular economy. In particular, this paper not only supplements the field of supply chain performance research with evidence from CDW resource utilization, but it also expands the field of circular economy development research from the perspective of information sharing.

To sum up, this paper focuses on the impact of recyclers’ information sharing on the performance of the construction waste resource utilization supply chain and focuses on answering the following three questions: First, how will recyclers’ information sharing affect the decisions and profits of the construction and demolition waste resource utilization supply chain? Second, in the construction and demolition waste resource utilization supply chain, how can a recycler benefit from information sharing? Third, how can a remanufacturer stimulate recyclers’ information sharing?

In the context of information asymmetry and information sharing, this paper studies the impact of recyclers’ sharing their market demand forecasting information on the performance of the construction and demolition waste resource utilization supply chain. This paper mainly uses the Bayesian Nash equilibrium theory in order to reveal the impact of the information sharing decision of the recycler in the construction and demolition waste resource utilization supply chain and it investigates the conditions of information sharing from a recycler in the construction and demolition waste resource utilization supply chain. At the same time, when the recycler is unwilling to share their information, a coordination mechanism was designed in order to encourage the recycler to share their information, in order to achieve a win–win situation with the remanufacturer.

The rest of this paper is structured as follows. Section 2 provides a relevant literature review; Section 3 presents a description of the problem and the associated assumptions; Section 4 establishes and solves the CDW resource utilization supply chain decision under the recyclers’ information sharing and information non-sharing conditions; Section 5 provides an analysis of this conditional model; Section 6 provides numerical and sensitivity analyses and, finally, Section 7 summarizes the conclusions and limitations of this paper.

## 2. Literature Review

In this section, this paper reviews the previous literature which pertains to three relevant aspects, as shown in Table 1. Firstly, this paper reviews the related research and latest progress of CDW management from the perspective of supply chain operation. Second, the definition and related literature on the dual-channel reverse supply chain (DRSC) are comprehensively reviewed. Finally, the research on the impact of information sharing on the supply chain’s performance and the research on the information sharing strategies of information holders are reviewed.

### 2.1. CDW Management from the Perspective of Supply Chain Operation

The existing research on CDW management from the perspective of supply chain operations can be divided into two categories: closed-loop supply chains and reverse supply chains. 

#### 2.1.1. Closed-Loop Supply Chain Aspects 

Li et al. [7] studied the behavioral decision-making problem of a closed-loop supply chain of CDW consisting of manufacturers, recyclers and retailers and pointed out that the concept of closed-loop supply chain management is very suitable for CDW management. Su et al. [22] built a closed-loop supply chain for CDW consisting of remanufacturers, manufacturers and recyclers in order to study the incentive mechanism of CDW recycling under the condition of information asymmetry. It should be noted that the information asymmetry in their research specifically refers to a moral hazard, which is not the same concept as the demand-related information that is investigated in the present paper. 

#### 2.1.2. Reverse Supply Chain Aspects

Hu et al. [23] proposed a CDW reverse supply chain consisting of manufacturers, recyclers and contractors. Based on this, they analyzed a dynamic game strategy for CDW recycling with learning and reference effects. Su et al. [24] developed a reverse supply chain of CDW consisting of contractors, recyclers and landfills in order to study the charging decisions for recyclers and landfills under different rights structures. 

The above studies have fully proved that it is feasible to solve the problem of CDW management from the perspective of supply chain operation and the concept of supply chain management is suitable for application to CDW management.

Existing studies have studied CDW from the perspective of closed-loop supply chain operations, but these studies have not considered the role of information sharing [38,39]. In this paper, we investigate the impact of recyclers’ information sharing on the performance of CDW resource utilization supply chains from a microscopic perspective. In the actual operation of the CDW supply chain, the recycling and remanufacturing (resource utilization) of CDW and the sales of CDW-based remanufactured products are two relatively independent processes. Therefore, it is more appropriate to study the CDW resource utilization supply chain from the perspective of a reverse supply chain operation.

Although the above-mentioned scholars have conducted in-depth research on CDW management from the perspective of a supply chain operation, they are all limited to offline recycling channels. They did not consider the related issues in the DRSC with the introduction of online recycling channels. However, in reality, there are many cases where remanufacturers open online recycling channels. Therefore, this paper introduces the reverse supply chain of online-and-offline dual recycling channels into CDW management for the first time.

### 2.2. DRSC

The dual-channel recycling model was first proposed in a study of closed-loop supply chains. Giri et al. [25] proposed a closed-loop supply chain model involving manufacturers, retailers and third parties, with manufacturers conducting their recycling activities through their own e-tail channels and third-party channels. Liu et al. [26] studied the pricing and reverse channel selection decision problems that exist in a closed-loop supply chain and found that a dual-channel recycling model is the optimal choice for manufacturers. It was not long before dual-channel reverse supply chains were discussed in articles in mainstream journals. Feng et al. [27] first introduced the concept of online recycling channels into the traditional reverse supply chain in 2017 and they constructed profit models for remanufacturers and recyclers under centralized decision-making and decentralized decision-making according to the different preferences of consumers. Chen et al. [28] first defined a DRSC in 2018 as a supply chain model in which online and offline recycling channels coexist. So far, the DRSC has been clearly explained: it is a two-level reverse supply chain system that is composed of remanufacturers and recyclers, in which the remanufacturers carry out recycling activities through dual online and offline channels.

So far, scholars have carried out research on the contract design, pricing strategy and network design of DRSC. Wu et al. [29] conducted research on the DRSC contract coordination problem considering the service level and they then established an effective revenue-sharing contract. Jin et al. [30] analyzed the influence of the power structure on the pricing decisions and profits of remanufacturers and recyclers in a DRSC. By incorporating consumers’ preference for online recycling channels into the system, Li and Wu [31] conducted research on the strategic network design of a DRSC and constructed a detailed quantitative model for network design optimization.

Although the above-mentioned scholars have conducted in-depth research on the decision-making of DRSC, the impact of demand information sharing has been neglected. However, in the DRSC (i.e., a CDW resource utilization supply chain) in the context of the construction industry, the uncertainty of the market’s demand information greatly hinders the scientific decision-making of the remanufacturers, which has a further serious impact on supply chain performance. Therefore, the impact of market demand information sharing on the supply chain of CDW resource utilization cannot be ignored and should be fully researched and revealed.

### 2.3. Information Sharing

With the continuous development of the field of supply chain management, the impact of demand information sharing on the supply chain has been increasingly recognized and investigated by the academic community. The research that is related to this paper includes the influence of information sharing on supply chain performance and the influence of the decision-making of information holders in a supply chain on information sharing.

#### Aspects of the Impact of Information Sharing on Supply Chain Performance

Lee et al. [32] explored how to quantify the benefits of sharing more demand information in a two-tier supply chain system consisting of retailers and manufacturers. They demonstrated that information sharing leads to better reductions in the supply chain’s costs and inventory levels. Cheng and Wu [33] considered the impact of information sharing on the inventory and expected costs in a two-tier supply chain with multiple retailers and found that the manufacturers’ expected costs and inventory levels decreased as the level of information sharing increased. From the above literature, one can conclude that information sharing can improve the performance of the supply chain. However, these related studies are based on the perspective of a positive supply chain. Although Wang et al. [34] came to a similar conclusion from the perspective of a dual-channel closed-loop supply chain, it has not yet been revealed whether the same conclusion can be drawn for the DRSC of CDW resource utilization supply chain.

Scholars usually hold two views on the decision-making of information holders in the supply chain regarding information sharing. The traditional view holds that the information holders will always suffer a loss of benefits due to their information sharing and that they will not share their information voluntarily. For example: Li [35] analyzed the impact of vertical information sharing on the profit and social benefits of the supply chain in a two-level supply chain that was composed of a manufacturer and multiple retailers and their results showed that the retailers never share their market demand information. Huang et al. [36] considered the value of information sharing in a dual-channel closed-loop supply chain consisting of manufacturers, retailers and collectors and found that the retailers’ information sharing would not increase or even reduce their own profits. Another view is that, in some cases, information holders will benefit from information sharing. For example: Guan et al. [37] explored the problem of sharing demand information in two competing supply chains and they found that the retailers would voluntarily share demand information if the manufacturer’s service investment was more efficient. This paper will further investigate this issue in the CDW resource utilization supply chain.

None of the above studies have examined CDW management from the perspective of DRSC operations. In addition, no scholars have studied the impact of demand information sharing on the DRSC. Therefore, the main contributions of this paper have two aspects. First, while there have been studies on CDW management from a supply chain operational perspective, these studies were not conducted in the context of a DRSC and did not take into account the issue of information sharing. However, these problems do in fact exist and the research that is presented within this paper aims to solve these problems. Secondly, according to the characteristics of the CDW resource utilization supply chain, a Stackelberg game model was constructed and the Bayesian equilibrium solution and expected profit under the two conditions of information sharing or not sharing were obtained for a comparative analysis. This paper then summarizes the rules that recyclers should follow in their decision-making regarding information sharing as remanufacturers’ environmental responsibility investment efficiency changes. In previous research on the dual-channel reverse supply chain, scholars have often ignored the impact of environmental responsibility investment efficiency changes on information sharing decisions and corporate profits. This also becomes an important contribution of this paper to the theoretical development of this field. Throughout the analysis of this paper, the aim is to provide a decision-making reference for the government and construction industry enterprises.

## 3. Problem Description and Associated Assumptions

**Figure 1:**
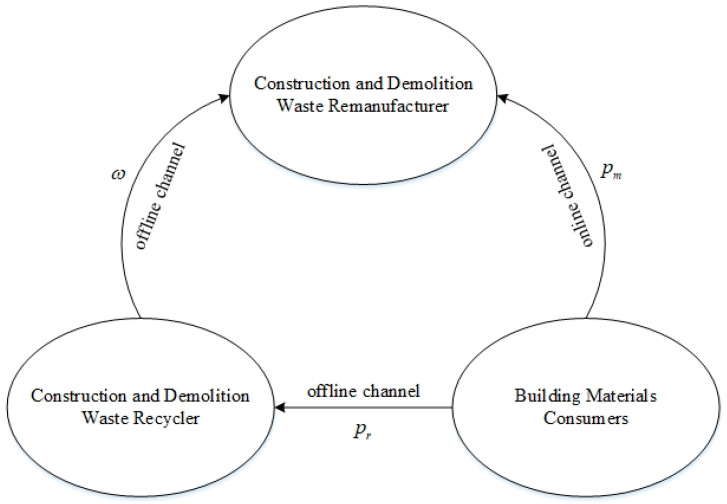
Game model.

Consider a two-level reverse green supply chain system consisting of a remanufacturer and a CDW recycler. Among them, the remanufacturer recycles CDW through both online and offline channels; that is, the remanufacturer directly recycles CDW from consumers through online channels and recycles CDW from the recycler through offline channels; the recycler only recycles CDW through offline channels. The formed game model is shown in Figure 1.

The remanufacturer recycles CDW from consumers and the recycler at different recycling prices and the recycler recycles CDW from consumers at a recycling price. As the leader of this game, the remanufacturer has a certain level of environmental responsibility; the recycler forecasts the market demand information and decides whether to share this information with the remanufacturer. This study benchmarks the information non-sharing model in order to analyze the impact of recyclers’ information sharing on the supply chain performance of CDW resource utilization. The notation, model assumptions, information structure and decision-making process of the recycler and remanufacturer game that is used in the study are described below (Table 2).

### 3.1. Model Assumptions

(1) In this study, remanufacturers are the leaders of the Stackelberg game and the CDW recyclers are the followers in this game [40,41].
(2) It is assumed that the common knowledge of the remanufacturer and recycler include the potential market demand a. In addition to the recycler’s market demand forecast information f is their private information, the recycler has the right to decide whether to share this information with the remanufacturer. The rest of the information is also common knowledge for the remanufacturer and recycler [42,43].
(3) This paper mainly examines the recycling pricing of recyclers and remanufacturers in the CDW resource utilization supply chain and does not consider the sales pricing of the remanufactured products. Therefore, separate from the CDW recycling prices $p_{r}$, $p_{m}$ and ω as decision variables, it is assumed that the market sales price of CDW remanufactured products is a constant p.
(4) The CDW that is recovered by the remanufacturer through offline channels is fully provided by the recycler and all of the CDW that is recovered through any means can be used for remanufacturing [44,45]. There is no difference in quality between the remanufactured products that are produced by the remanufacturer using CDW and new products that are produced from the raw materials [42,46]; that is, the CDW that is recycled through dual channels does not affect the quality of offline new product construction materials. However, these remanufactured products are superior to new products in terms of their environmental and economic performance [47].
(5) In the offline recycling channel, the recycler can recycle CDW through their well-established recycling network; in the online recycling channel, the remanufacturer can contact consumers in order for them to recycle their CDW through the Internet. Obviously, in the production and operation processes, neither the recycler nor the remanufacturer has a fixed cost input. Therefore, this study does not consider the fixed costs of the recycler and the remanufacturer.

### 3.2. Information Structure

We have assumed that the potential market demand is random, that is, $a=a_{0}+e$, where *a*_0_ is the determined part of the potential market demand and e is the uncertain part of the potential market demand, E(e)=0, var(e)=μ. It was assumed that the forecast value of the potential market demand of a CDW recycler is f=a+ε. Where ε is the forecast error, E(ε)=0, var(ε)=υ and the random variables e and ε are independent of each other. According to relevant literature research [37,43,48], there are:

(1) $$\left\{ \begin{array}{l} E\left(a|f\right)=\frac{v}{u+v}a_{0}+\frac{u}{u+v}f\equiv A\\ E[(f-a_{0})²]=\mu +\upsilon \end{array}\right.$$

We let t=μ/(μ+υ) be the prediction accuracy of a CDW recycler’s information, t∈(0,1). The larger the t, the more accurate the recycler’s market demand forecast; whereas the smaller the t, the less accurate the recycler’s market demand forecast. As such, there is $E\left(a|f\right)=(1-t)a_{0}+tf\equiv A$.

### 3.3. Game Order

**Figure 2:**
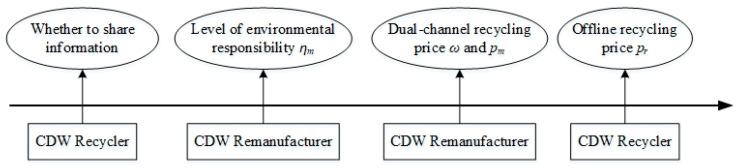
Game order.

In CDW resource utilization supply chains, the remanufacturer is the leader of the Stackelberg game and the recycler is the follower. The game order (as shown in Figure 2) is: first, the recycler decides whether to share their demand forecast information with the remanufacturer; secondly, the remanufacturer decides their own environmental responsibility level according to whether the recycler shares their demand forecast information and the degree of the optimism or pessimism of that demand forecast information; thirdly, the remanufacturer predicts the recycler’s pricing situation and then determines the online and offline recycling prices for recycling CDW and, finally, the recycler decides the recycling price for their own recycling of CDW, based on the remanufacturer’s pricing.

## 4. Model Building and Solving

In the CDW resource utilization supply chain, the demand functions of offline channels and online channels are:

(2) $$\left\{ \begin{array}{l} q_{r}=\phi a+b+p_{r}-\theta p_{m}+k_{m}\eta_{m}\\ q_{m}=\left(1-\phi \right)a+b+p_{m}-\theta p_{r}+k_{m}\eta_{m} \end{array}\right.$$

### 4.1. Recycler Information Is Not Shared

In the CDW resource utilization supply chain, if recyclers do not share their market demand forecast information, the expected profit decision models of the remanufacturer and the recycler are:

(3) $$\left\{ \begin{array}{l} \underset{p_{m},\omega ,\eta_{m}}{\max}E\left(\prod \begin{array}{c} IN\\ M \end{array}\right)=E\left(\begin{array}{l} \left(p-\omega \right)\left(\phi a_{0}+b+p_{r}-\theta p_{m}+k_{m}\eta_{m}\right)+\\ \left(p-p_{m}\right)\left(\left(1-\phi \right)a_{0}+b+p_{m}-\theta p_{r}+k_{m}\eta_{m}\right)-\frac{1}{2}c_{m}\eta_{m}{}^{2} \end{array}\right)\\ \underset{p_{r}}{\max}E\left(\prod \begin{array}{c} IN\\ R \end{array}|f\right)=E\left(\left(\omega -p_{r}\right)\left(\phi A+b+p_{r}-\theta p_{m}+k_{m}\eta_{m}\right)|f\right) \end{array}\right.$$

We solved the optimal ω, $p_{m}$, $p_{r}$ and $\eta_{m}$, without sharing information, as:

(4) $$\left\{ \begin{array}{l} \omega^{IN^{*}}=\frac{4\left(\left(b+p\left(-1+\theta \right)\right)\left(1+\theta \right)+\left(\theta +\phi -\theta \phi \right)a_{0}\right)c_{m}+\left(2p\left(3+4\theta +\theta^{2}\right)-\left(2+\theta \right)\left(-1+2\phi \right)a_{0}\right)k_{m}^{2}}{2\left(1+\theta \right)\left(4\left(-1+\theta \right)c_{m}+\left(3+\theta \right)k_{m}^{2}\right)}\\ p_{m}^{IN^{*}}=\frac{4\left(\left(b+p\left(-1+\theta \right)\right)\left(1+\theta \right)+\left(1+\left(-1+\theta \right)\phi \right)a_{0}\right)c_{m}+\left(2p\left(3+4\theta +\theta^{2}\right)+\left(-1+2\phi \right)a_{0}\right)k_{m}^{2}}{2\left(1+\theta \right)\left(4\left(-1+\theta \right)c_{m}+\left(3+\theta \right)k_{m}^{2}\right)}\\ p_{r}^{IN^{*}}=\frac{\left\{\begin{array}{l} 2\left(\left(1+\theta \right)\left(-b\left(-3+\theta \right)+\left(-1+\theta \right)\left(p+p\theta -2A\phi \right)\right)+\left(-2\theta \left(-1+\phi \right)+\phi +\theta^{2}\phi \right)a_{0}\right)c_{m}\\ +\left(\left(3+4\theta +\theta^{2}\right)\left(2p-A\phi \right)+\left(3+2\theta -3\phi +\theta^{2}\phi \right)a_{0}\right)k_{m}^{2} \end{array}\right\}}{2\left(1+\theta \right)\left(4\left(-1+\theta \right)c_{m}+\left(3+\theta \right)k_{m}^{2}\right)}\\ \eta_{m}^{IN^{*}}=-\frac{\left(\left(3+\theta \right)\left(b+p-p\theta \right)+\left(2+\left(-1+\theta \right)\phi \right)a_{0}\right)k_{m}}{4\left(-1+\theta \right)c_{m}+\left(3+\theta \right)k_{m}^{2}} \end{array}\right.$$

The optimal expected profits of the remanufacturer and recycler are:

(5) $$\left\{ \begin{array}{l} E\left(\prod \begin{array}{c} IN^{*}\\ M \end{array}\right)=\frac{\left\{-2\left(\begin{array}{l} {\left(b+p-p\theta \right)}^{2}\left(3+4\theta +\theta^{2}\right)+2\left(1+\theta \right)\left(b+p-p\theta \right)\left(2+\left(-1+\theta \right)\phi \right)a_{0}\\ +\left(2+4\left(-1+\theta \right)\phi +\left(3-4\theta +\theta^{2}\right)\phi^{2}\right)a_{0}^{2} \end{array}\right)c_{m}+{\left(1-2\phi \right)}^{2}a_{0}^{2}k_{m}^{2}\right\}}{4\left(1+\theta \right)\left(4\left(-1+\theta \right)c_{m}+\left(3+\theta \right)k_{m}^{2}\right)}\\ E\left(\prod \begin{array}{c} IN^{*}\\ R \end{array}\right)=\frac{\left\{\begin{array}{l} 4{\left(-1+\theta \right)}^{2}\left(4t\mu \phi^{2}+{\left(b+p-p\theta +\phi a_{0}\right)}^{2}\right)c_{m}^{2}+4\left(-1+\theta \right)\\ \left(2t\left(3+\theta \right)\mu \phi^{2}+\left(-1+2\phi \right)a_{0}\left(b+p-p\theta +\phi a_{0}\right)\right)c_{m}k_{m}^{2}+\left(t{\left(3+\theta \right)}^{2}\mu \phi^{2}+{\left(1-2\phi \right)}^{2}a_{0}^{2}\right)k_{m}^{4} \end{array}\right\}}{4{\left(4\left(-1+\theta \right)c_{m}+\left(3+\theta \right)k_{m}^{2}\right)}^{2}} \end{array}\right.$$

### 4.2. Recycler Information Sharing

In the CDW resource utilization supply chain, if the recycler and the remanufacturer share their demand forecast information, the expected profit decision models of the remanufacturer and the recycler are:

(6) $$\left\{ \begin{array}{l} \underset{p_{m},\omega }{\max}E\left(\prod \begin{array}{c} \mathrm{IS}\\ M \end{array}|f\right)=E\left(\begin{array}{l} \left(p-\omega \right)\left(\phi A+b+p_{r}-\theta p_{m}+k_{m}\eta_{m}\right)+\\ \left(p-p_{m}\right)\left(\left(1-\phi \right)A+b+p_{m}-\theta p_{r}+k_{m}\eta_{m}\right)-\frac{1}{2}c_{m}\eta_{m}^{2}|f \end{array}\right)\\ \underset{p_{r}}{\max}E\left(\prod \begin{array}{c} IS\\ R \end{array}|f\right)=E\left(\left(\omega -p_{r}\right)\left(\phi A+b+p_{r}-\theta p_{m}+k_{m}\eta_{m}\right)|f\right) \end{array}\right.$$

We solved the optimal ω, $p_{m}$, $p_{r}$ and $\eta_{m}$ under the condition of information sharing as:

(7) $$\left\{ \begin{array}{l} \omega^{IS^{*}}=\frac{\left\{\begin{array}{l} 4\left(b\left(1+\theta \right)+p\left(-1+\theta^{2}\right)+A\left(\theta +\phi -\theta \phi \right)\right)c_{m}+\\ \left(2p\left(3+4\theta +\theta^{2}\right)-A\left(2+\theta \right)\left(-1+2\phi \right)\right)k_{m}^{2} \end{array}\right\}}{2\left(1+\theta \right)\left(4\left(-1+\theta \right)c_{m}+\left(3+\theta \right)k_{m}^{2}\right)}\\ p_{m}^{IS^{*}}=\frac{4\left(A+\left(b+p\left(-1+\theta \right)\right)\left(1+\theta \right)+A\left(-1+\theta \right)\phi \right)c_{m}+\left(2p\left(3+4\theta +\theta^{2}\right)+A\left(-1+2\phi \right)\right)k_{m}^{2}}{2\left(1+\theta \right)\left(4\left(-1+\theta \right)c_{m}+\left(3+\theta \right)k_{m}^{2}\right)}\\ p_{r}^{IS^{*}}=\frac{\left\{\begin{array}{l} -2\left(-p\left(-1+\theta \right){\left(1+\theta \right)}^{2}+b\left(-3-2\theta +\theta^{2}\right)+A\left(2\theta \left(-1+\phi \right)-3\phi +\theta^{2}\phi \right)\right)c_{m}\\ +\left(2p\left(3+4\theta +\theta^{2}\right)-A\left(3+2\theta \right)\left(-1+2\phi \right)\right)k_{m}^{2} \end{array}\right\}}{2\left(1+\theta \right)\left(4\left(-1+\theta \right)c_{m}+\left(3+\theta \right)k_{m}^{2}\right)}\\ \eta_{m}^{IS^{*}}=-\frac{\left(\left(3+\theta \right)\left(b+p-p\theta \right)+A\left(2+\left(-1+\theta \right)\phi \right)\right)k_{m}}{4\left(-1+\theta \right)c_{m}+\left(3+\theta \right)k_{m}^{2}} \end{array}\right.$$

The optimal expected profits of the remanufacturer and recycler are:

(8) $$\left\{ \begin{array}{l} E\left(\prod \begin{array}{c} IS^{*}\\ M \end{array}\right)=\frac{\left\{-2\left(\begin{array}{l} {\left(b+p-p\theta \right)}^{2}\left(3+4\theta +\theta^{2}\right)+2\left(1+\theta \right)\left(b+p-p\theta \right)\\ \left(2+\left(-1+\theta \right)\phi \right)a_{0}+\left(\begin{array}{l} 2+4\left(-1+\theta \right)\phi +\\ \left(3-4\theta +\theta^{2}\right)\phi^{2} \end{array}\right)\left(t\mu +a_{0}^{2}\right) \end{array}\right)c_{m}+{\left(1-2\phi \right)}^{2}\left(t\mu +a_{0}^{2}\right)k_{m}^{2}\right\}}{4\left(1+\theta \right)\left(4\left(-1+\theta \right)c_{m}+\left(3+\theta \right)k_{m}^{2}\right)}\\ E\left(\prod \begin{array}{c} IS^{*}\\ R \end{array}\right)=\frac{\left\{\begin{array}{l} 4{\left(-1+\theta \right)}^{2}\left({\left(b+p-p\theta \right)}^{2}+2\left(b+p-p\theta \right)\phi a_{0}+\phi^{2}\left(t\mu +a_{0}^{2}\right)\right)c_{m}^{2}-4\left(-1+\theta \right)\\ \left(1-2\phi \right)\left(t\mu \phi +a_{0}\left(b+p-p\theta +\phi a_{0}\right)\right)c_{m}k_{m}^{2}+{\left(1-2\phi \right)}^{2}\left(t\mu +a_{0}^{2}\right)k_{m}^{4} \end{array}\right\}}{4{\left(4\left(-1+\theta \right)c_{m}+\left(3+\theta \right)k_{m}^{2}\right)}^{2}} \end{array}\right.$$

## 5. Model Analysis

**Proposition** **1.** *The impact of information sharing strategies on decision variables:*

*When* $A>a_{0}$, *there are* $\omega^{IN^{*}}>\omega^{IS^{*}}$, $p_{m}{}^{IN^{*}}>p_{m}{}^{IS^{*}}$, $p_{r}{}^{IN^{*}}>p_{r}{}^{IS^{*}}$, $\eta_{m}^{IN^{*}}>\eta_{m}^{IS^{*}}$;

*When* $A<a_{0}$, *there are*$\omega^{IN^{*}}<\omega^{IS^{*}}$, $p_{m}{}^{IN^{*}}<p_{m}{}^{IS^{*}}$, $p_{r}{}^{IN^{*}}<p_{r}{}^{IS^{*}}$, $\eta_{m}^{IN^{*}}<\eta_{m}^{IS^{*}}$.

Proposition 1 shows that if the recycler’s forecast value of market demand is greater than the average potential market demand (i.e., $A>a_{0}$), then information sharing will enable the remanufacturer and recycler to set lower recycling prices (including the three recycling prices ω, $p_{r}$ and $p_{m}$ in both the online and offline channels) and a lower level of environmental responsibility. If the recycler’s forecast of the market demand is less than the average potential market demand (i.e., $A<a_{0}$), then information sharing will lead to higher recycling prices for the remanufacturer and recycler (including the three recycling prices ω, $p_{r}$ and $p_{m}$ in both the online and offline channels) and a higher level of environmental responsibility. This is because when the market demand is higher in the information that is obtained by the remanufacturer it will formulate lower ω and $p_{m}$ values and increase the profit. At the same time, due to the decrease in ω, the recycler will decrease their $p_{r}$ in order to avoid a loss of their own profits. From Proposition 1, it can be seen that the size of the predicted value of the recycler’s demand information and the decision of whether to share the information greatly affects the remanufacturer’s decision about the recycling price and the level of their environmental responsibility. Therefore, a remanufacturer should strive to obtain information in order to make more scientific and beneficial decisions.

**Proposition** **2.** *The influence of the information sharing strategy on the remanufacturer’s profit: Recyclers’ information sharing is beneficial to the increase in the remanufacturer’s profit*.

Proposition 2 shows that information that is shared by the recycler is always beneficial to the increase in a remanufacturer’s profits; that is, the value of the recycler’s market demand forecast information to the remanufacturer is positive. This is because the remanufacturer can make more scientific decisions on recycling prices and environmental responsibility levels based on their own experience after obtaining the demand forecast information, so as to obtain maximum profits. For the remanufacturer, in order to maximize their profits, they should strive to obtain market demand information. If the recycler is willing to share their forecast information, a win–win situation can be achieved. If the recycler is unwilling to share their forecast information, the remanufacturer can incentivize them to share by paying information fees or by other measures.

**Proposition** **3.** *The impact of information sharing strategies on recycler profits*:

(1) *When* $c_{m}\in [c_{m1},c_{m2}]$, *information sharing will increase the profit of the recycler*;
(2) *When* $c_{m}\in [0,c_{m1}]\cup [c_{m2},1]$*, the impact of information sharing on the profit of the recycler is not significant.*

Proposition 3 indicates that the impact of information sharing on the profits of the recycler is not linear. That is, when the efficiency of the environmental responsibility investment of the remanufacturer is in an appropriate range, information sharing increases the profits of the recycler and, in this situation, the remanufacturer and recycler achieve a win–win situation. However, when the efficiency of the environmental responsibility investment of the remanufacturer is too low or too high, the impact of information sharing on the profits of the recycler is not significant. Furthermore, in this situation, the recycler has no incentive to share their demand forecast information with the remanufacturer for free. When the remanufacturer’s environmental responsibility investment efficiency is at a high level, it means that the effect of the remanufacturer’s environmental responsibility level is more obvious. It can be seen from the demand function that the recycling volume of CDW in both online and offline channels increased in this situation. At this time, both the recycler and remanufacturer benefit from the environmental responsibility investment of the remanufacturer. At the same time, if the remanufacturer obtains market demand information, when the efficiency of the remanufacturer’s environmental responsibility investment is appropriate, the impact of the environmental responsibility level is enhanced and limited capital investment can significantly increase the recycling volume. The profit increase for the recycler due to the environmental responsibility investment of the remanufacturer is much greater than their profit loss due to their information sharing; overall increasing the total profit of the recycler. However, when the remanufacturer’s environmental responsibility investment is too high or too low, the impact of their environmental responsibility level is weakened and the recycling volume and market share will not be significantly affected. In this situation, the profit of the recycler is not affected by their information sharing.

**Proposition** **4.** *The impact of information sharing on the profits of CDW resource utilization supply chains*:

(1) *When* $c_{m}\in [0,c_{m3}]$, *information sharing will reduce the profit of the supply chain; when* $c_{m}\in [c_{m3},c_{m4}]$, *information sharing will increase the profit of the supply chain; when* $c_{m}\in [c_{m4},1]$*, information sharing will increase the profit of the supply chain slightly but not significantly.*
(2) *When* $\theta \in [\theta_{1},\theta_{2}]$*, information sharing will increase the profit of the supply chain*.
(3) *When* $\phi \in [0,\phi_{1}]$*, information sharing will increase the profit of the supply chain; when* $\phi \in [\phi_{1},1]$*, information sharing will reduce the profit of the supply chain*.

Proposition 4 shows that when the investment efficiency of the environmental responsibility is low, the impact of information sharing on the overall profit of the supply chain is not significant. When the investment efficiency of the environmental responsibility is high, information sharing is beneficial to increasing the overall profit of the supply chain. However, when the investment efficiency of the environmental responsibility is too high, information sharing is not conducive to the increase in the overall profit of the supply chain. When the competition intensity between the online and offline channels is relatively high, information sharing is beneficial to increasing the overall profit of the supply chain. When the competition intensity between online and offline channels is too high or too low, information sharing has no significant impact on the overall profit of the supply chain. This is because when the remanufacturer’s environmental responsibility investment efficiency is too high, the information sharing increases the remanufacturer’s profit but is not enough to make up for the loss of the recycler’s profit, resulting in a decrease in the overall profit of the supply chain. When the remanufacturer’s environmental responsibility investment efficiency is high, information sharing can improve the profits of both the remanufacturer and the recycler, thereby increasing the overall profit of the supply chain. When the remanufacturer’s environmental responsibility investment efficiency is low, information sharing has no significant impact on the remanufacturer’s profit, but it slightly increases the recycler’s profit, which increases the overall profit of the supply chain. When the competition intensity between the online and offline channels is high, information sharing can enable the remanufacturer to make more scientific decisions about their level of environmental responsibility, thereby increasing the recycling volumes of the online and offline channels. On the one hand, it makes up for the loss of the recycler due to their information sharing and on the other hand, it increases the profit of the remanufacturer, thereby increasing the overall profit of the supply chain. When the offline channel’s share is small, the profit that is gained by the remanufacturer due to the recycler’s information sharing is much greater than the profit that is lost by the recycler, so the overall profit of the supply chain increases. When the market share of the offline channels is large, the profits that are gained by the remanufacturer due to the recycler’s information sharing are not enough to make up for the profits that are lost by the recycler due to their information sharing, so the overall profits of the supply chain are reduced.

The above propositions analyze the impact of the recycler’s information sharing on the performance of the CDW resource utilization supply chain. From propositions 1 and 2, it can be seen that information sharing from a recycler is always beneficial to the increase in the profits of the remanufacturer. However, when the efficiency of the environmental responsibility investment is too high or too low, information sharing will not increase the profits of the recycler themselves. At this time, the recycler has no incentive to share their demand forecast information for free. In order to seek more profits, the remanufacturer can encourage the recycler to share their demand information by paying certain information fees. The information fee W, which is paid, needs to meet the following conditions:

(9) $$\left\{ \begin{array}{l} E\left(\prod \begin{array}{c} IS^{*}\\ M \end{array}\right)-E\left(\prod \begin{array}{c} IN^{*}\\ M \end{array}\right)-W\geq 0\\ E\left(\prod \begin{array}{c} IS^{*}\\ R \end{array}\right)-E\left(\prod \begin{array}{c} IN^{*}\\ R \end{array}\right)+W\geq 0 \end{array}\right.$$

Thus have $E\left(\prod \begin{array}{c} IN^{*}\\ R \end{array}\right)-E\left(\prod \begin{array}{c} IS^{*}\\ R \end{array}\right)\leq W\leq E\left(\prod \begin{array}{c} IS^{*}\\ M \end{array}\right)-E\left(\prod \begin{array}{c} IN^{*}\\ M \end{array}\right)$.

**Proposition** **5.** *Conditions for recyclers’ information sharing*:

(1) $c_{m}\in [c_{m1},c_{m2}]$;
(2) $c_{m}\in [0,c_{m1}]\cup [c_{m2},1]$*and*$W\in [-\Delta \prod {}_{R},\Delta \prod {}_{M}]$.

Proposition 5 states that the recycler always shares their demand forecast information with the remanufacturer if the remanufacturer’s environmental responsibility investment efficiency is appropriate. If the investment efficiency of the remanufacturer’s environmental responsibility is too high or too low and the remanufacturer’s payment of information fees meets certain conditions, the recycler will also share their information with the remanufacturer. At this time, a win–win situation is achieved. Profits for both the remanufacturer and the recycler are boosted by this information sharing. Proposition 5 provides a reference for the decision of the remanufacturer. That is, when the recycler has no incentive to share their forecast information, the remanufacturer can choose to pay information fees or they can take measures such as assuming appropriate environmental responsibility investment efficiency in order to encourage the recycler to share their information.

## 6. Numerical Simulation

### 6.1. Numerical Analysis

This section uses a numerical analysis model and the correctness of the conclusions to analyze the impact of recyclers’ information sharing on the supply chain performance in the CDW resource utilization supply chain. According to the existing research, we determined the values of the following parameters: $a_{0}=100$ [49], ϕ=0.5 [50]. Ten experts from the construction waste resource utilization supply chain field (including professors, doctors and masters) discussed the reliability and importance of the remaining parameters through face-to-face discussions and confirmed the following: p=30, b=100, t=0.6, $k_{m}=0.4$, θ=0.4, $c_{m}=0.5$ and μ=100,000. It is worth noting here that despite the high environmental value of CDW its market recycling demand is volatile. Currently, the time cost for consumers to sell CDW may be greater than the income that these sales generate. When most consumers choose a CDW disposal method, it is not necessarily their first choice to sell to a recycler or remanufacturer. Therefore, the market demand is not stable. This variance not only expresses the degree of deviation of the sample from the mean, but also reveals the degree of fluctuation within the sample. It can also be understood that the variance represents the expectation of the fluctuation of the sample. Therefore, based on the uncertainty of the market demand in reality, after expert discussion, the value of μ was set to 100,000.

**Figure 3:**
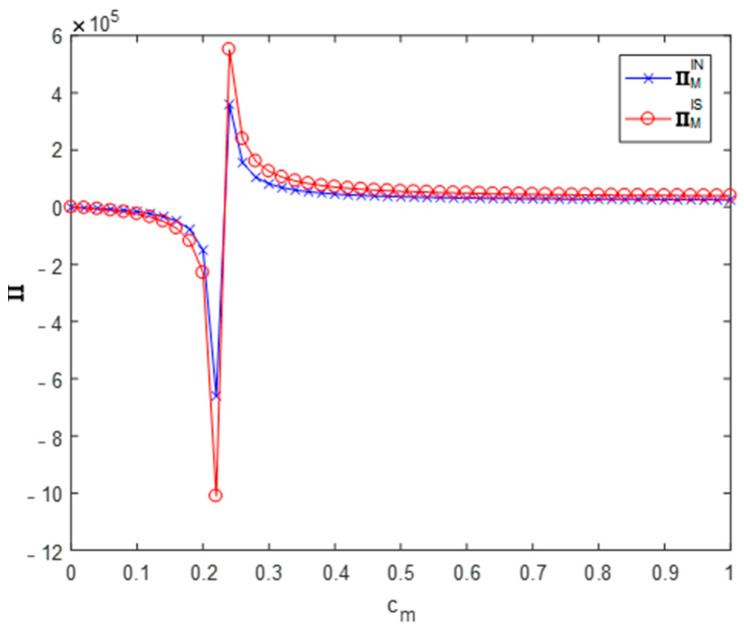
Effect on the remanufacturer’s profit.

Setting θ=0.4, ϕ=0.5 and $c_{m}\in [0,1]$ allowed us to get the influence of the recycler’s information sharing strategy on the profit of the remanufacturer (as shown in Figure 3).

Figure 3 shows that, in a profitable situation, the recycler sharing their forecast information with the remanufacturer always benefits the remanufacturer’s profits ($\prod \begin{array}{c} IS\\ M \end{array}>\prod \begin{array}{c} IN\\ M \end{array}$).

**Figure 4:**
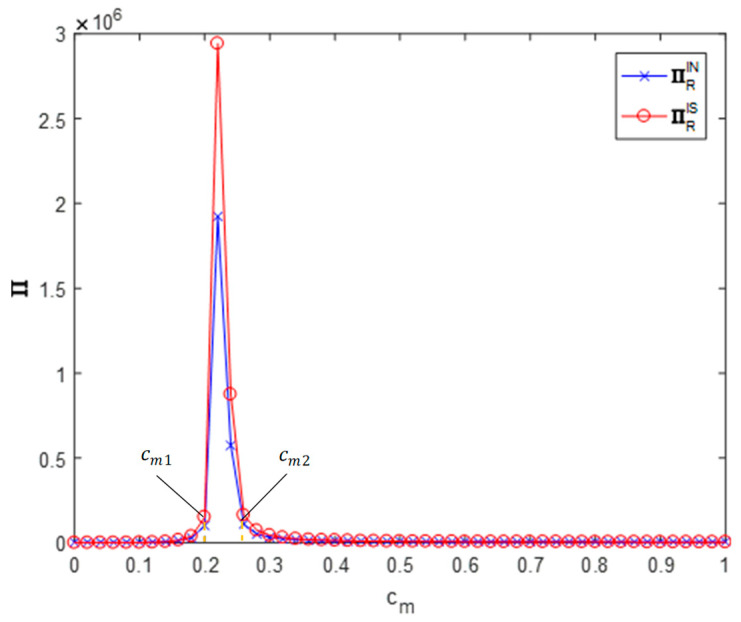
Impact on recycler profits.

By setting θ=0.4, ϕ=0.5 and $c_{m}\in [0,1]$, the impact of the recycler’s information sharing strategy on the profit of the recycler can be obtained (as shown in Figure 4).

In the region $c_{m}\in [c_{m1},c_{m2}]$, the efficiency of environmental responsibility investment is in a high range, the profit of the recycler increases first and then decreases with the efficiency of the environmental responsibility investment and information sharing increases the profit of the recycler ($\prod \begin{array}{c} IS\\ R \end{array}>\prod \begin{array}{c} IN\\ R \end{array}$). In this situation, the recycler has an incentive to share their demand forecast information with the remanufacturer for free. In the region $c_{m}\in [0,c_{m1}]\cup [c_{m2},1]$, the efficiency of the environmental responsibility investment is too high or too low so information sharing has no significant impact on the profits of the recycler. At this point the recycler is unprofitable and has no incentive to share their demand forecast information with the remanufacturer.

**Figure 5:**
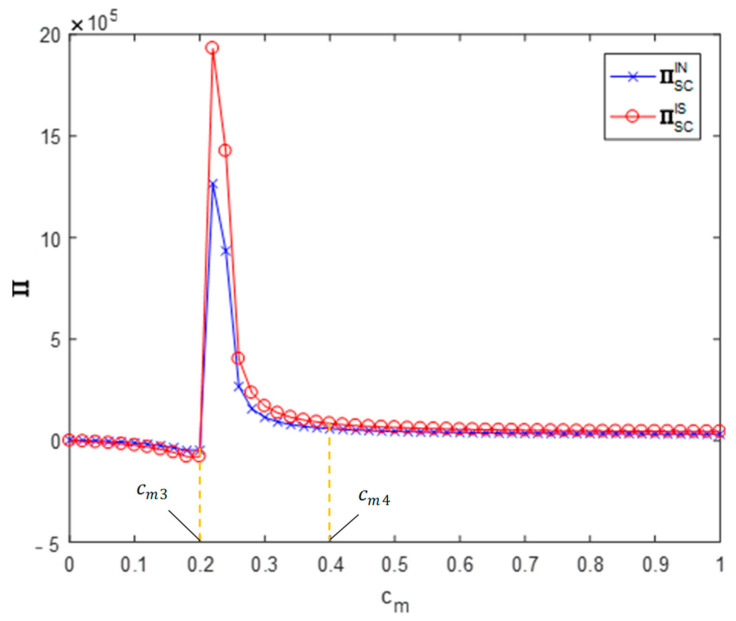
Impact on supply chain profits.

**Figure 6:**
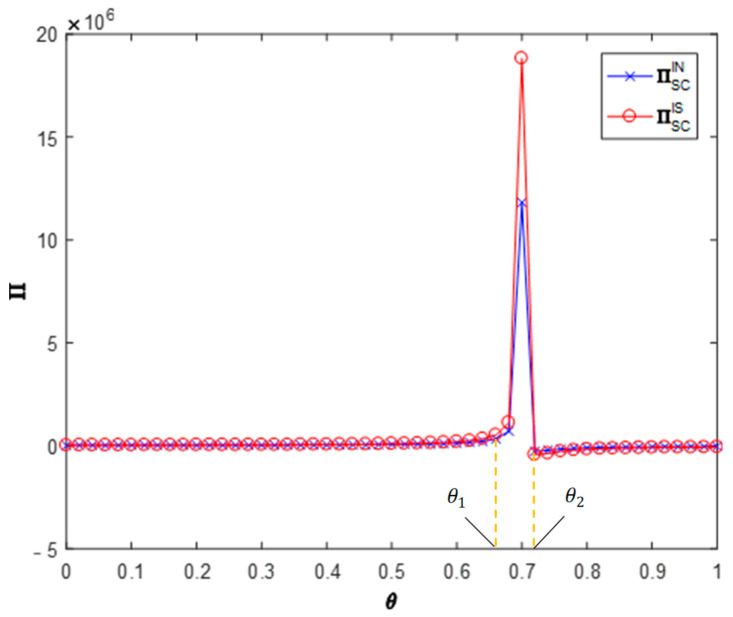
Impact on supply chain profits.

**Figure 7:**
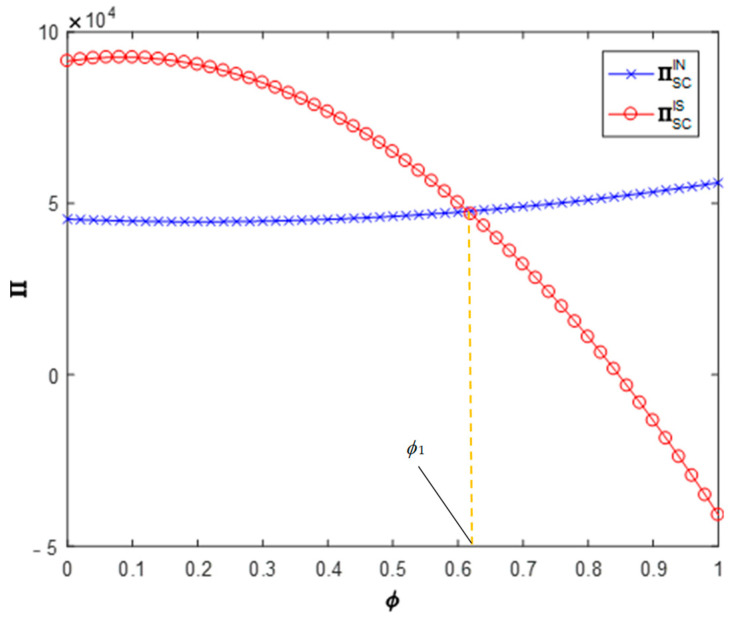
Impact on supply chain profits.

Figure 5, Figure 6 and Figure 7 analyze the impact of recyclers’ information sharing on the profit of the supply chain. Letting θ=0.4, ϕ=0.5 and $c_{m}\in [0,1]$, the impact of the recyclers’ information sharing strategy on the overall profit of the CDW resource utilization supply chain with the change of the efficiency of environmental responsibility investment can be obtained (as shown in Figure 5). According to these values, information sharing in area $c_{m}\in [0,c_{m3}]$ will reduce the overall profit of the supply chain ($\prod \begin{array}{c} IS\\ SC \end{array}<\prod \begin{array}{c} IN\\ SC \end{array}$). Information sharing in region $c_{m}\in [c_{m3},c_{m4}]$ will increase the overall profit of the supply chain ($\prod \begin{array}{c} IS\\ SC \end{array}>\prod \begin{array}{c} IN\\ SC \end{array}$). In the area $c_{m}\in [c_{m4},1]$, information sharing makes the overall profit of the supply chain slightly increase but not significantly. Letting ϕ=0.5, $c_{m}=0.5$ and θ∈[0,1], the impact of the recyclers’ information sharing strategy on the overall profit of the CDW resource utilization supply chain with the change of the cross-recycling price influence coefficient can be obtained (as shown in Figure 6). Accordingly, in the region $\theta \in [0,\theta_{1}]$, information sharing has no significant impact on the overall profit of the supply chain. Information sharing in region $\theta \in [\theta_{1},\theta_{2}]$ will increase the overall profit of the supply chain ($\prod \begin{array}{c} IS\\ SC \end{array}>\prod \begin{array}{c} IN\\ SC \end{array}$). In the region $\theta \in [\theta_{2},1]$, information sharing has no significant impact on the overall profit of the supply chain. Letting $c_{m}=0.5$, θ=0.4 and ϕ∈[0,1], the impact of the recyclers’ information sharing strategy on the overall profit of the CDW resource utilization supply chain with the change of the offline recycling channel market share can be obtained (as shown in Figure 7). Per these values, information sharing in the region $\phi \in [0,\phi_{1}]$ will increase the overall profit of the supply chain ($\prod \begin{array}{c} IS\\ SC \end{array}>\prod \begin{array}{c} IN\\ SC \end{array}$) and information sharing in area $\phi \in [\phi_{1},1]$ will reduce the overall profit of the supply chain ($\prod \begin{array}{c} IS\\ SC \end{array}<\prod \begin{array}{c} IN\\ SC \end{array}$). It can be seen that the numerical simulation results are consistent with the above conclusions.

### 6.2. Sensitivity Analysis

In the numerical simulation analysis in the previous section, the influence of the cross-recycling price influence coefficient θ, market share of offline recycling channels ϕ and environmental responsibility investment cost coefficient $c_{m}$ on the profits of the remanufacturer and the recycler under the conditions of no information sharing and information sharing was studied. This section further analyzes the sensitivity of the fixed p, t, b and $k_{m}$ in Section 6.1 according to the analysis steps of the literature [51,52,53,54].

**Figure 8:**
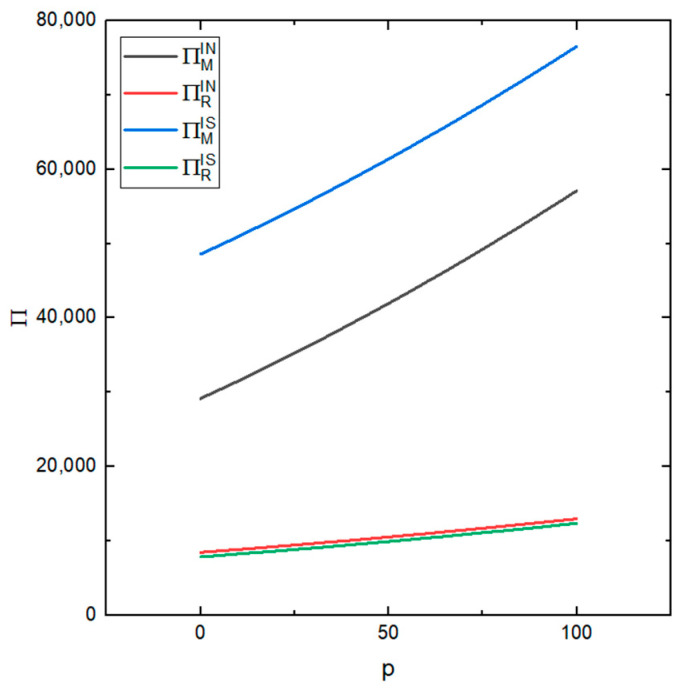
The effect of different values of p on the profits of the recycler and remanufacturer.

Figure 8 shows the influence of the market selling price *p* of remanufactured products under different values on the profits of the remanufacturer and the recycler under the two conditions (whether the recycler’s information is shared or not). It can be seen from the figure that the remanufacturer is more sensitive to the change in this value, while the recycler is less sensitive to this change. But, as can be seen from the figure, as *p* increases the profits of the recycler and the remanufacturer will also increase. This shows that the higher the market price of remanufactured products, the higher the profits of the remanufacturer and the recycler. Raising the selling price of the remanufactured products can effectively improve the profits of the recycler and the remanufacturer and increase the total profits of the supply chain.

**Figure 9:**
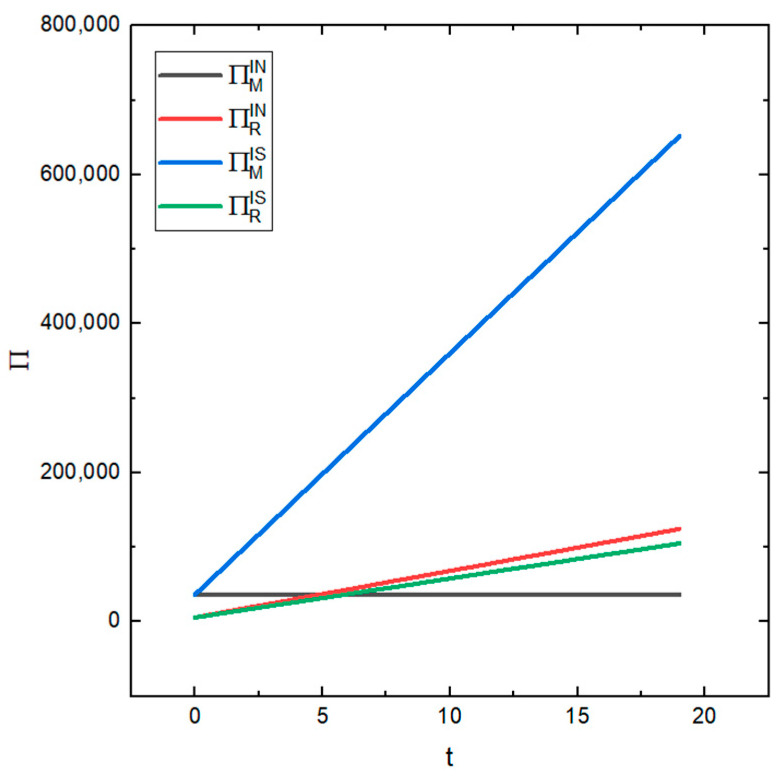
The effect of different values of t on the profits of the recycler and remanufacturer.

Figure 9 shows the influence of different values of the prediction accuracy t of the recycler information on the profits of the manufacturer and the recycler under the two conditions of whether the recycler information is shared or not. It can be seen from the figure that the remanufacturer is more sensitive to t when the recycler’s information is shared than when the information is not shared. With the increase in t, the profit of the remanufacturer under the condition of information sharing will also increase, while the profit of the remanufacturer under the condition of no information sharing is almost unchanged. The recycler’s sensitivity to t is greater under the condition of no information sharing than under the condition of information sharing. As t increases, the profit of the recycler will also increase. This shows that the degree of the information prediction’s accuracy has an impact on the profit of the recycler in the two cases and it has an impact on the profit of the remanufacturer under the information sharing condition. Improving the accuracy of the recycler’s information prediction can also improve the total profit of the supply chain.

**Figure 10:**
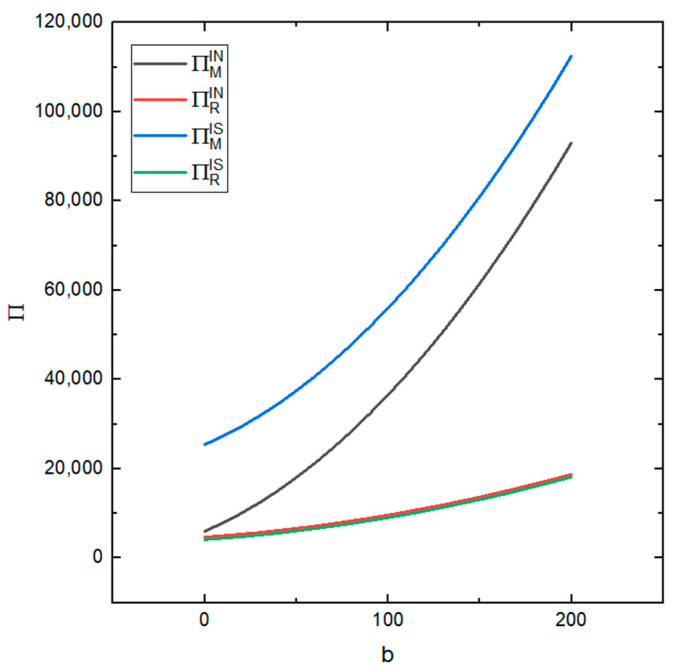
The impact of different values of b on the profits of the recycler and remanufacturer.

Figure 10 shows the impact of consumers’ green development concept b on the profits of the remanufacturer and the recycler under the two conditions (whether or not the recycler’s information is shared or not). It can be seen from the figure that the remanufacturer’s profit is more sensitive to *b* than the recycler’s profit and it is more sensitive to *b* in both cases, i.e., the recycler sharing their information and the recycler not sharing their information. That is, with the increase in *b*, the profit of the remanufacturer also increases and, although the profit of the recycler also increases, the recycler’s increase is small. This shows that improving consumers’ green development concept can increase the total profit of the supply chain.

**Figure 11:**
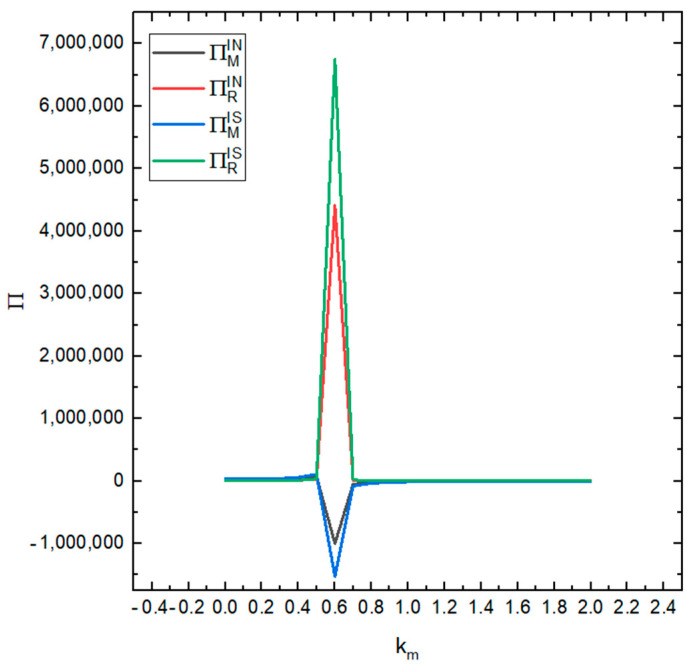
The influence of different values of $k_{m}$ on the profits of the recycler and remanufacturer.

Figure 11 shows the impact of the consumer environmental protection sensitivity coefficient *k* on the profits of the remanufacturer and the recycler under the two conditions (the recycler sharing their information or not). It can be seen from the figure that the profit of the recycler in both cases (sharing the information and not sharing the information) increases slowly with the increase in the value of $k_{m}$. When $k_{m}$ is between 0.5 and 0.6, the profit surges. When the value is 0.6, the profit reaches its maximum value, it drops sharply between 0.6–0.7 and then slowly decreases. The profit of the remanufacturer is in the range of 0.5–0.7 and the profit changes from a positive value to a negative value and then back to a positive value. On the whole, the total profit of the supply chain has increased. Especially when the value of $k_{m}$ is around 0.6, the profit is at its largest.

## 7. Conclusions and Implications

### 7.1. Conclusions

In the current construction industry market environment, the sharing of the recycler’s demand forecast information is still an important factor affecting the decision-making and performance of enterprises in the CDW resource utilization supply chain and it has always attracted the attention of many scholars and practitioners. However, few existing studies discuss it in depth. In this regard, this paper uses the game theory method to study the impact of recyclers’ information sharing on the performance of the CDW resource utilization supply chain. Firstly, the supply chain decision-making model under the two situations of recyclers sharing their information and recyclers not sharing their information was constructed and then, based on the recycler information non-sharing model, the Bayesian equilibrium solution and expected profit were obtained, compared and analyzed. Finally, the conditions under the condition of the recycler sharing their information were given and a numerical simulation was used to analyze the influence of the important parameters on the performance of the supply chain and a sensitivity analysis of the parameters was carried out. The specific findings of the study can be summarized as follows:

First, when the recycler’s demand forecast is pessimistic, the information sharing will allow the remanufacturer to receive the signal of the market downturn. At this time, remanufacturers will take measures to improve their own environmental responsibility level in order to stimulate the market to expand the demand for recycling CDW and further increase their own profits. When the recycler’s demand forecast is more optimistic, the information sharing will enable the remanufacturer to receive a signal of market activity. This will enable remanufacturers to reduce their own environmental responsibility level, in order to obtain greater benefits with limited capital investment by reducing their environmental responsibility costs.

Second, in the CDW resource utilization supply chain, because recyclers are closer to the consumer market, they can obtain market information and predict market demand more effectively. If recyclers share their demand forecast information with remanufacturers free of charge, remanufacturers will make scientific and reasonable decisions on pricing and their own environmental responsibilities; as such their profits will always increase due to information sharing.

Third, whether recyclers have an incentive to share their demand forecast information with the remanufacturer and whether the recycler should charge for the information depend on the efficiency of the remanufacturer’s environmental responsibility investments. When the environmental responsibility investment efficiency of the remanufacturer is in a high range, the recycler will obtain higher income due to their information sharing. In this situation, the recycler is motivated to share their information for free; in other situations, their information will not be shared for free.

Fourth, for the CDW resource utilization supply chain as a whole, the impact of information sharing by recyclers depends on the intensity of the competition among the channels, the market share of offline recycling channels and the efficiency of the recyclers’ environmental responsibility investment. In most cases, recyclers’ information sharing is beneficial to the overall profit increase in the CDW resource utilization supply chain or it has no significant impact on the profit. However, when the efficiency of the environmental responsibility investment of remanufacturers is too high or the market share of offline recycling channels is large, recyclers’ information sharing will cause losses to the entire CDW resource utilization supply chain.

Fifth, when recyclers are reluctant to share their information, remanufacturers can take two effective measures to encourage the recyclers to share their information, thereby increasing their own profits and improving the performance of the entire CDW resource utilization supply chain. Namely: (1) remanufacturers can properly improve the efficiency of their environmental responsibility investment, so that the recyclers can increase their income due to their information sharing, thereby urging the recyclers to share their information and (2) the remanufacturer can pay a certain information fee to the recycler, so that the profit that is lost by the recycler due to their information sharing can be compensated, thereby encouraging the recycler to share their demand forecast information.

### 7.2. Implications

This research enables companies that are engaged in CDW recycling and remanufacturing business to have a more comprehensive and rich understanding of the impact of recyclers’ information sharing behavior on supply chain performance. This study also provides important management implications for the government and companies in the CDW resource utilization supply chain, namely:

(1) For remanufacturers, there is always a benefit from the sharing of information in the CDW resource utilization supply chain. Therefore, remanufacturers should strive to obtain demand forecast information from recyclers. When the cost of obtaining the information is less than the increase in income, that is, when the marginal income is greater than zero, a certain fee can be paid to the recycler in order to obtain their demand forecast information and improve the remanufacturer’s own profit. Alternatively, the remanufacturer can assume the appropriate environmental responsibility investment efficiency to achieve a win–win situation with the recycler, prompting the recycler to share their demand forecast information.
(2) For recyclers, when information is shared, the level of the environmental responsibility of the remanufacturer can be affected by controlling the degree of their optimism or pessimism within their market demand forecast information, thereby affecting the efficiency of the remanufacturer’s environmental responsibility investment, so as to increase their own profits.
(3) For the government, in order to promote the efficient and sustainable development of the CDW resource utilization industry, relevant policies should be introduced in order to stimulate the overall profit of the supply chain. Specifically, corresponding policies should be introduced that require the remanufacturer’s environmental responsibility input, so as to avoid the remanufacturer’s environmental responsibility input cost coefficient being too low. In order to achieve an effective level of macro-control, the government must also encourage the remanufacturer to use online channels to recycle CDW and adopt policies such as raising the taxes on offline CDW recycling channels in order to avoid offline recycling channels occupying too much market share.

Although this study has involved analysis which aims to examine the impact of recyclers’ information sharing on the supply chain of CDW resource utilization, it has certain limitations. For example, although the game model that is constructed in this paper theoretically explains the impact of information sharing on the performance of the CDW resource utilization supply chain, it has not been proved by actual cases. This paper only studies a CDW resource utilization supply chain that is composed of one remanufacturer and one recycler. In reality, there are many remanufacturers and recyclers. In the future, the researchers may consider recyclers’ information sharing strategies in the case of multiple remanufacturers or multiple recyclers. In addition, this paper only considers the situation in which the recycler makes demand forecasts and the researchers could go on to study the situation in which the recycler and the remanufacturer make demand forecasts at the same time.

## Figures and Tables

**Table 1:** Research related to construction and demolition waste and supply chain management and information sharing.

Research Topics	Dimensions	Sosurce Papers
CDW management from a supply chain operational perspective	Behavioral decision-making in closed-loop supply chains of CDW	[7]
	Recycling incentives in closed-loop supply chains of CDW under asymmetric information	[22]
	Recycling dynamic game strategy in the reverse supply chain of CDW	[23]
	Charging decisions in the reverse supply chain of CDW under different rights structures	[24]
DRSC	Closed-loop supply chain for recycling activities through e-tail and third-party channels	[25]
	Pricing and reverse channel selection decision problems in closed-loop supply chains	[26]
	Reverse supply chain including online recycling channels	[27]
	Supply chain model with coexistence of online and offline recycling channels	[28]
	Contract design, pricing strategy and network design	[29,30,31]
Information sharing	The impact of information sharing on supply chain performance	[32,33,34]
	The decisions of information holders in the supply chain on information sharing behavior	[35,36,37]

**Table 2:** Model parameters.

Parameter	Definition
a	CDW market potential recycling demand. a ≥ 0
$q_{m}$	Online CDW recycling demand. $q_{m}$ ≥ 0
$q_{r}$	Recycling demand for offline CDW. $q_{r}$ ≥ 0
ϕ	Market share of offline recycling channels. 0 < ϕ<1
b	Consumers’ green development concept.
θ	Cross-recycling price influence coefficient and 0 < θ < 1, wherein the larger the θ, the more intense the competition between channels
$k_{m}$	Consumer environmental protection sensitivity coefficient, which represents the sensitivity of consumers to the level of environmental responsibility of the remanufacturer.
$c_{m}$	Environmental responsibility investment cost coefficient, which represents the efficiency of the remanufacturer’s environmental responsibility investment.
p	Market price of remanufactured CDW products. p > 0
W	Information payment fee. W > 0
$\prod \begin{array}{c} Y\\ X \end{array}$	The profit of X under the Y model. X=M, R, SC represent Remanufacturer, recycler and supply chain, respectively; Y=IN, IS represent recycler information not sharing and information sharing, respectively.
ω	Recycling prices for CDW recovered by remanufacturer from recycler in offline recycling channels (Decision variables).
$p_{r}$	Recycling prices for CDW recovered by recycler from consumers in offline recycling channels (Decision variables).
$p_{m}$	Recycling prices for CDW recovered directly from consumers by remanufacturer in online recycling channels (Decision variables).
$\eta_{m}$	Remanufacturer’s level of environmental responsibility (Decision variables).

## Data Availability

Not applicable.
